# Defining the Core Proteome of the Chloroplast Envelope Membranes

**DOI:** 10.3389/fpls.2013.00011

**Published:** 2013-02-06

**Authors:** Stefan Simm, Dimitrios G. Papasotiriou, Mohamed Ibrahim, Matthias S. Leisegang, Bernd Müller, Tobias Schorge, Michael Karas, Oliver Mirus, Maik S. Sommer, Enrico Schleiff

**Affiliations:** ^1^Institute of Molecular Cell Biology of Plants, Goethe UniversityFrankfurt, Germany; ^2^Institute of Pharmaceutical Chemistry, Goethe UniversityFrankfurt, Germany; ^3^Department of Biology I, Ludwig-Maximilians-UniversityMunich, Germany; ^4^Center of Membrane Proteomics, Goethe UniversityFrankfurt, Germany; ^5^Cluster of Excellence ‘Macromolecular Complexes’, Goethe UniversityFrankfurt, Germany

**Keywords:** membrane proteome, plant proteomics, chloroplast membrane proteins, mass spectrometry, envelope membrane proteome approach comparison

## Abstract

High-throughput protein localization studies require multiple strategies. Mass spectrometric analysis of defined cellular fractions is one of the complementary approaches to a diverse array of cell biological methods. In recent years, the protein content of different cellular (sub-)compartments was approached. Despite of all the efforts made, the analysis of membrane fractions remains difficult, in that the dissection of the proteomes of the envelope membranes of chloroplasts or mitochondria is often not reliable because sample purity is not always warranted. Moreover, proteomic studies are often restricted to single (model) species, and therefore limited in respect to differential individual evolution. In this study we analyzed the chloroplast envelope proteomes of different plant species, namely, the individual proteomes of inner and outer envelope (OE) membrane of *Pisum sativum* and the mixed envelope proteomes of *Arabidopsis thaliana* and *Medicago sativa*. The analysis of all three species yielded 341 identified proteins in total, 247 of them being unique. 39 proteins were genuine envelope proteins found in at least two species. Based on this and previous envelope studies we defined the core envelope proteome of chloroplasts. Comparing the general overlap of the available six independent studies (including ours) revealed only a number of 27 envelope proteins. Depending on the stringency of applied selection criteria we found 231 envelope proteins, while less stringent criteria increases this number to 649 putative envelope proteins. Based on the latter we provide a map of the outer and inner envelope core proteome, which includes many yet uncharacterized proteins predicted to be involved in transport, signaling, and response. Furthermore, a foundation for the functional characterization of yet unidentified functions of the inner and OE for further analyses is provided.

## Introduction

The characterization of a single protein function is associated with an enumeration of different features. Some of these features are the subcellular localization of the protein, its interaction with other proteins, co- or post-translational modifications as well as its (enzymatic) activity. With the growing number of sequenced genomes, the “proteome,” as sum of all proteins in an entire cell or cellular (sub-)compartment, becomes important for the understanding of cellular function (Wilkins et al., 1996; James, 1997). The mass spectrometric analysis of complete cellular proteomes still remains difficult, especially in the highly compartmentalized eukaryotic cells. Furthermore, proteomes are dynamic and change in response to different stimuli. They include different splice forms and post-translationally modified proteins in different abundances. Thus, different technical approaches have been developed to accommodate the complexity of a proteome (e.g., Karas and Hillenkamp, 1988; Aebersold and Mann, 2003), especially to study the subcellular localization of membrane proteins as a complementary approach to the complete cell proteome analyses (van Wijk, 2000; Millar et al., 2009).

This complexity of eukaryotic cells leads us to focus on the proteome of chloroplast, which are organelles essential for different metabolic pathways like photosynthesis, and biosynthesis of fatty acids or amino acids. These organelles contain several thousand different proteins and the majority of which is cytosolically synthesized and has to be translocated across the envelope membranes (Leister, 2003; Schleiff and Becker, 2011).Thereby, the proteome of the organelle as such (Zabrouskov et al., 2003; Kleffmann et al., 2004) or of subfractions like the thylakoid lumen (Peltier et al., 2002), the thylakoid membranes (Eichacker et al., 2004; Friso et al., 2004), the stroma (Goulas et al., 2006; Peltier et al., 2006), plastoglobules (Ytterberg et al., 2006), or the envelope membranes (Schleiff et al., 2003b; Bräutigam and Weber, 2009) have been analyzed in the past. The current knowledge on the proteomic content of chloroplasts has been deposited in several independent databases like PLPROT (Kleffmann et al., 2006) or AT_CHLORO (Ferro et al., 2010). However, especially the analysis of the envelope and more specifically the inner envelope (IE) and outer envelope (OE) membrane proteome is still a challenging task due to the hydrophobicity of membrane proteins (Eichacker et al., 2004). More specifically, the dissection of the IE and OE membrane proteome is still very poorly supported by direct proteomic studies (Ferro et al., 2003; Schleiff et al., 2003b).

The determination of a protein’s localization is a very important tool for experimental guidance. In here, we aimed at the determination of a reliable proteome of the OE and IE membranes of chloroplasts. To this end, we comparatively analyzed the overall envelope proteomes of the model species *Arabidopsis thaliana* and *Medicago sativa*. To substantiate our findings, we individually analyzed IE and OE membranes of *Pisum sativum*, the only plant to date, for which the separation of both can be achieved (Ferro et al., 2003; Schleiff et al., 2003b). We chose the genetic model *A. thaliana* by its comprehensive genome and transcriptome data available (see, e.g., The Arabidopsis Information Resource, TAIR10; Lamesch et al., 2012). The legume *P. sativum* was chosen, as it is the model plant for biochemical analyses of chloroplast function (see, e.g., Franssen et al., 2012). Due to the paucity of data, the recently sequenced and closely related *M. sativa* was used to substantiate our findings for *P. sativum*.

The identified proteins in these plant species were compared to each other and to the publicly available datasets of previous studies. We identified a total of 247 different proteins, of which – based on comparisons with other studies – 191 were assigned as putative envelope proteins. To our surprise, only 27 of these were found in all studies. Based on intersection and cross-contamination analysis of available previous studies, we were able to reliably assign 50/49 proteins as outer/inner membrane-localized, while at least 37 additional proteins in the mixed envelope fractions can be assigned as envelope proteins as well, but not reliably to a specific membrane.

## Results and Discussion

### Chloroplast proteome analyses

We analyzed the chloroplast proteomes with focus on the envelope membranes from three model plant species, namely *A. thaliana*, *P. sativum*, and *M. sativa*. We chose *A. thaliana* because of the availability of a comprehensive genome and many existing transcriptome data (e.g., The Arabidopsis Information Resource, TAIR10; Lamesch et al., 2012). Thus, the well annotated genome of *Arabidopsis* provides a solid base for the assignment of the identified inner and OE proteins. In turn, the legumes *P. sativum* and *M. sativa* are model plants for biochemical analyses of chloroplast function (e.g., Franssen et al., 2012), as well as crop plants. Using envelopes of different plant species allows the detection of proteins with different abundances. The varying achievable purity of the samples allows the detection of an additional different set of peptides.

**Figure 1:**
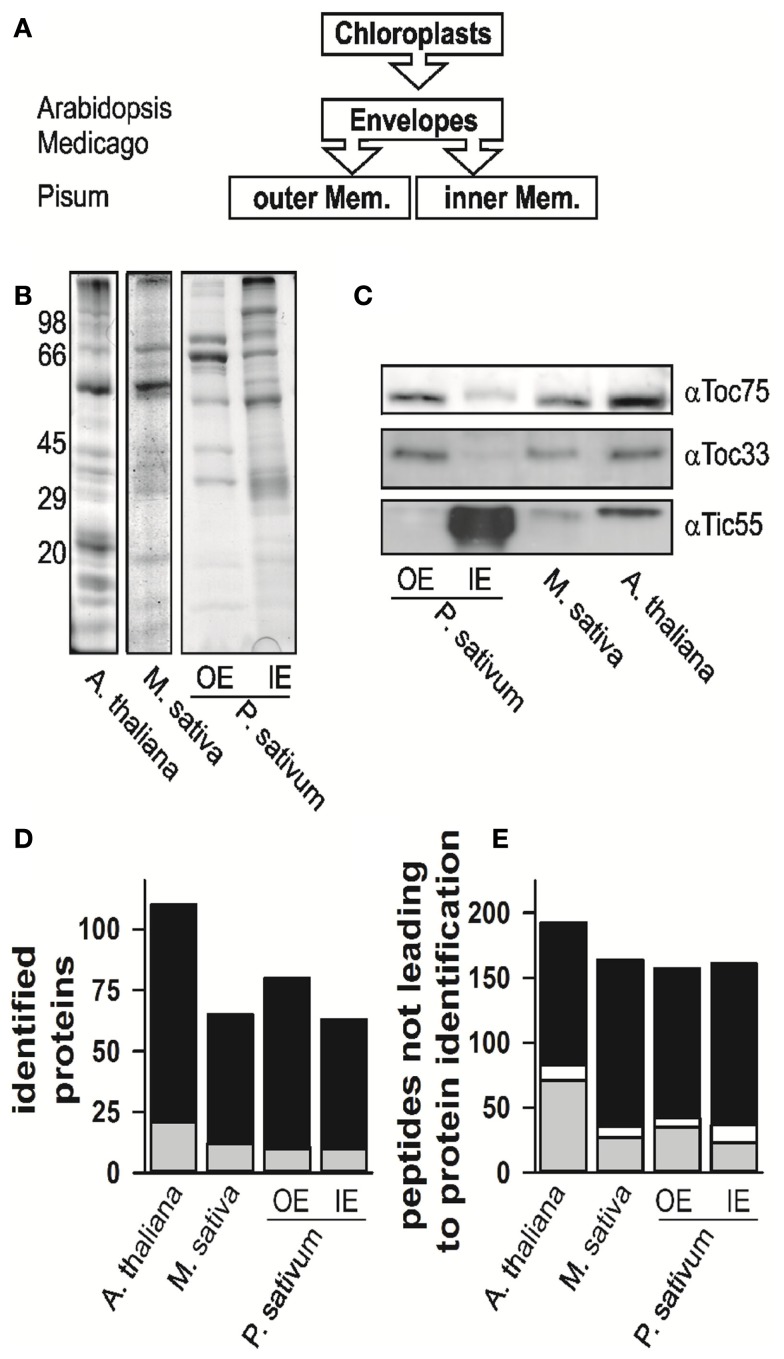
**The proteome analysis**. **(A)** Schematic representation of which fractions were isolated and analyzed. The different species are indicated for the envelope fraction results of six independent replicates, three after trypsin and three after elastase digestion were combined. **(B)** The fractions of mixed envelope of *A. thaliana* and *M. sativa* as well as the outer (OE) and inner envelope (IE) membrane of *P. sativum* were subjected to SDS-PAGE analysis followed by Coomassie Blue staining. The migration of the molecular weight standard is indicated on the left. **(C)** The purity of the fractions in **(B)** was assessed by Western blotting using indicated antibodies. **(D)** Numbers of proteins identified in the according fractions by MALDI nano-LC-MS/MS and the two digestion methods indicated. Gray indicates the portion for which more than one AGI was assigned for one protein family, in white the portion where more than one isoform was specifically identified for one protein, black indicates the portion for which one AGI was assigned. **(E)** Numbers of peptides not assigned by MALDI nano-LC-MS/MS and BLAST assignment. Gray indicates the portion of peptides, which were assigned to one amino acid sequence only, whereas white indicates the portion of peptides, which were assigned to various proteins, black indicates the portion of peptides, which were not assigned at all.

We isolated and subfractionated chloroplasts to analyze the envelope proteomes (Figures 1A,B). The enrichment of the obtained fractions was assessed by Western blotting using specific antibodies (Figure 1C). The analysis confirmed the enrichment of inner and outer membrane proteins in the mixed envelope fractions of *A. thaliana* and *M. sativa*, the mixed envelope fractions could not be further separated. In contrast, separation of envelope membranes in the IE and OE from *P. sativum* chloroplasts has been established previously (e.g., Schleiff et al., 2003a,b). Subsequently, the distinct fractions were analyzed by mass spectrometry.

The proteomes of all envelope membranes were analyzed by MALDI nLC-MS/MS (Table S14 in Supplementary Material) yielding in total 110 proteins in *A. thaliana* (Figure 1D, *Arabidopsis* EM, three independent isolations; Table S1 in Supplementary Material). In parallel, we identified 71 proteins in *M. sativa* (Figure 1D, *Medicago* EM, three independent isolations; Table S2 in Supplementary Material) and 124 different proteins in both membranes (87 IE; 73 OE) of *P. sativum* (Figure 1D, *Pisum* IE and *Pisum* OE, three independent isolations; Tables S3 and S4 in Supplementary Material).

**Figure A1:**
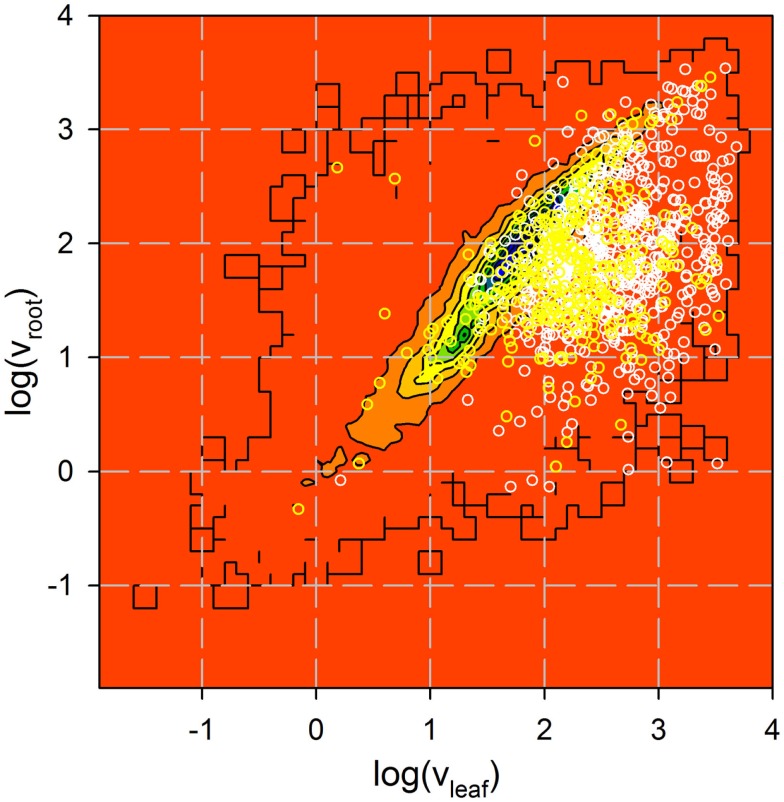
**Analysis of expression of genes coding for identified proteins**. Expression of 735 genes coding for proteins identified by more than one peptide (white) and 322 genes coding for proteins identified by one peptide only (yellow) in roots and leaves was analyzed by Affymetrix (Vojta et al., 2004). Shown is the Affymetrix value in logarithmic scale for all genes previously analyzed as contour plot (from low (red) to high (blue) number of genes identified with a certain expression distribution) and the distribution of the genes coding for the proteins identified as circles.

Our peptide-based assignment relies on a stringent BLAST search, where an identity >95% and no mismatch or gap was allowed. Only a single amino acid substitution with a residue of similar properties or a single undefined amino acid position was accepted (for details see Experimental Section). The BLAST search was combined with a bidirectional best BLAST hit search to assign the homologous sequences in *A. thaliana* to the proteins identified in *P. sativum* or *M. sativa* to render the assignment from different species comparable. To confirm that the peptide-based assignment is consistent with the expected chloroplast localization, we analyzed the expression of the corresponding genes with respect to leaves and roots (e.g., Vojta et al., 2004). Indeed, almost all genes coding for the identified proteins including those identified by a single peptide only are highly expressed in leaf tissue (Figure A1 in Appendix). AT3G45360 is the only exception identified by more than one peptide with an expression value smaller than 10 in leaves. However, this gene is annotated as a transposable element. Furthermore, almost all genes are equally high or higher expressed in leaves in comparison to roots. The only gene with a significantly higher expression in roots than in leaves is AT3G09260 identified in *A. thaliana*. It encodes a β-glucosidase annotated as Pyk10, which was identified in ER-bodies (Matsushima et al., 2003). Although the protein most likely represents a contamination of the sample, its overall expression pattern supports the peptide-based protein assignment approach.

While analyzing the data, a large number of the obtained peptides did not lead to an identification of a protein (Figure 1E, Tables S5–S7 in Supplementary Material). About 15–30% of these peptides mapped uniquely to a single sequence (in gray), while few peptides mapped to multiple sequences (in white). The large portion of peptides which remained unassigned (in black) might have three different reasons: (i) The choice of too stringent search parameters, (ii) contaminations of the samples, or (iii) the existence of natural variances of sequences in form of unknown splice variants or nucleotide polymorphisms of genes leading to alternative amino acid sequences. The analysis of this phenomenon, however, goes beyond the scope of this work.

### Comparison to other envelope membrane proteomic approaches

To establish a core envelope proteome we unified results of our and previous studies (Ferro et al., 2003, 2010; Froehlich et al., 2003; Bräutigam et al., 2008; Bräutigam and Weber, 2009). For that, we first assigned the *Arabidopsis* Genome Initiative (AGI) number of the closest homolog of *A. thaliana* to each of the proteins found in *M. sativa* and *P. sativum*. Combining our four data sets, we obtained 247 different proteins in total. The globally unified protein pool contains a total of 911 different proteins. Ferro et al. (2010) assigned their identified proteins according to the suborganellar (stroma, thylakoid, and envelope) localization, which we have used to assess the quality of our data (cross-contaminations from thylakoid and stroma). We defined four different categories (Table 1): Category I are proteins that were found in at least two studies but not in the stroma and thylakoid according to Ferro et al. (2010). Category II unites proteins, which were found in at least three studies but also in the stroma or thylakoid. Category III are proteins found in one study only, but exclusively in the envelope, and category IV are proteins found in less than three studies, but also in the stroma or thylakoid. The selection of three independent studies for category II as criterion takes into account that two studies each come from Bräutigam et al. (2008), Bräutigam and Weber (2009), and Ferro et al. (2003, 2010). For better visualization of the impact of our study we have marked the identified proteins of the categories as identified in here (a) or in previous studies (b).

**Table 1:** **Categories for the classification of envelope membrane proteins**.

Category	Mixed envelope fraction		Thylakoid or Stroma	Proteins identified in the given number of studies							
	This study	Others	Ferro et al. (2010)	6	5	4	3	2	1	SUM	OTH
Ia	+	At least one	−	27	12	25	17	18	–	99	11/8
Ib	−	At least two	−	–	5	11	41	75	–	132	20/4
IIa	+	At least two	+	3	14	11	16	–	–	44	0/0
IIb	−	At least three	+	–	2	9	17	–	–	28	2/0
IIIa	+	None	−	–	–	–	–	–	48	48	6/10
IIIb	−	One	−	–	–	–	–	–	298	298	68/46
IVa	+	Less than two	+	–	–	–	–	35	21	56	0/0
IVb	−	Less than three	+	–	–	–	–	53	153	206	16/0

*Given is the category defined in the text (column 1), protein identification by us (+, column 2) or by any other proteomic study (column 3 defines the required number of identifications), identification in the thylakoid or stroma (+, Ferro et al., 2010; column 4) and the number of proteins identified in 6, 5, 4, 3, 2, or 1 study (column 5–10) as well as the number of proteins in each category (column 11), and the number of proteins, which have been identified in other cellular fractions than chloroplasts as well based on PPDB (Sun et al., 2009)/SubaII (Heazlewood et al., 2007; column 12)*.

From our point of view the list of proteins of category I is most reliable, because there are no cross-contaminations via thylakoid and stroma and they are supported by previous studies. Proteins of categories II and III have to be confirmed experimentally first and proteins of category IV are considered to be not reliably assigned.

We noticed that only 30 proteins were identified in all six studies (categories Ia, IIa, Table 1), of which three have been identified in the stroma or thylakoid as well. In total, we found 231 proteins of category I. Additionally, we found 346 proteins of category III according to Ferro et al. (2010), which are not cross-contaminations of the stroma or thylakoid (Ferro et al., 2010). Hence, they might represent envelope proteins as well. However, as stated above, this conclusion should be challenged by biochemical approaches. The latter holds true for particularly 72 proteins of category II, which have been identified in envelope and in stroma or thylakoid. However, 262 proteins have been assigned to category IV.

Based on the PPDB and SubaII databases, we next analyzed whether proteins have been previously assigned to the mitochondrion, peroxisome, nucleus, ER, golgi, plasma membrane or cytosol, and not to the plastid (Table 1; Heazlewood et al., 2007; Sun et al., 2009). Accordingly, 31/12 proteins of category I were assigned to other cellular localizations according to PPDB/SubaII, respectively. In category II we found 2/0 proteins and in category III 74/56 proteins, respectively, which have been identified in cellular compartments other than chloroplasts. Thus, about 10 and 20% of the proteins assigned to category I or category III are found in other cellular compartments than the chloroplast. The low abundance of mislocalized proteins in category II might reflect that the proteome of the stroma and thylakoid (Ferro et al., 2010) has been established quite well. Nevertheless, the assignment of proteins in other organellar fractions does not necessarily mark them as false positive chloroplast proteins as (i) chloroplasts are the major organelles of plant cells and thus, contaminations of other fractions might exist and (ii) an increasing number of proteins are found to be dually localized (Carrie and Small, 2013).

### Comparison of the identified envelope proteomes of the different species

**Figure 2:**
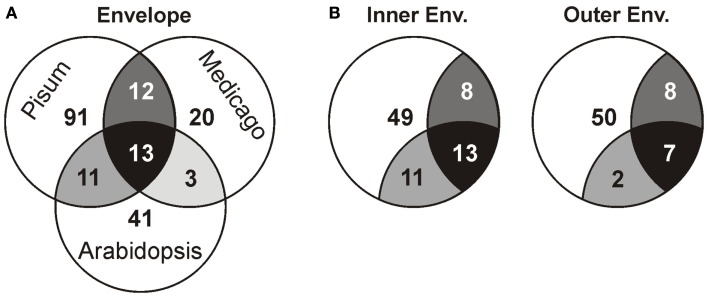
**Analysis of the different proteomes with respect to the putative localization**. **(A)** The overlap of the proteome determined for *A. thaliana*, *M. sativa*, and *P. sativum* mixed envelope was analyzed by MALDI nano-LC-MS/MS. The overlap is displayed by the shared regions of the circles in the Venn diagram. **(B)** The overlap of the proteome determined for *A. thaliana*, *M. sativa* mixed envelope and *P. sativum* inner (left) and outer (right) envelope was analyzed and the color code is taken from **(A)**.

Next, we compared our envelope proteomes obtained for the different analyzed plants with focus on proteins assigned to categories I–III (Table S8 in Supplementary Material). The 191 of total proteins assigned included 48 proteins identified in *M. truncatula*, 68 proteins in *A. thaliana*, and 127 proteins in *P. sativum*. Thirty-nine proteins were identified in at least two plant species, 13 of which were found in all three (Figure 2A; Table 2). Dissecting the protein set of *P. sativum* into OE and IE localized, revealed a total of 46 OE and 60 IE proteins. Twenty-one proteins were found in both fractions. We compared the OE and IE proteins separately with the identified envelope proteins of the other two plants (Figure 2B). This analysis shows that all 13 proteins identified in all species were also found in the IE, while 7 of them are also found in the OE. Similarly, all proteins found in the overlap between *P. sativum* and *A. thaliana* are found in the IE fraction (11), while the overlap with the *M. sativa* envelope contains four proteins (AT2G01320, AT4G32250, Toc64-III, and Toc132) specifically found in the OE of *P. sativum*.

**Table 2:** **Proteins in the envelope fraction of at least two species**.

AGI	Abbr.	*A. thaliana*	*M. sativa*	*P. sativum*			AGI	Abbr.	*A. thaliana*	*M. sativa*	*P. sativum*		
				OE	IE	M					OE	IE	M
*At1g03130*	PSAD-2	X	X		X		At3g26740	CCL	X				X
At1g06950	Tic110	X			X		At3g46740	Toc75-III	X	X			X
At1g08640	CJD1	X			X		At3g47520	MDH	X			X	
*At1g15820*	LHCB6	X	X		X		At3g63410	Iep37	X	X		X	
*At1g55670*	PSAG		X		X		At4g02510	Toc159	X	X			X
At1g65260	VIPP1		X		X		At4g15440	HPL1		X		X	
*At1g67090*	RBCS1A		X			X	*At4g20360*	RAB8d	X			X	
*At1g74470*	Unknown	X			X		At4g25450	NAP8	X			X	
At1g77590	LACS9		X			X	At4g32250	Unknown		X	X		
At2g01320	Unknown		X	X			*At4g32260*	Unknown	X	X			
At2g16640	Toc132		X	X			*At4g32770*	SDX1	X	X		X	
At2g24820	Tic55-II	X	X		X		At4g33350	Unknown		X			X
At2g28900	Oep16	X				X	At4g39460	SAMC1	X	X			
*At2g39730*	RCA	X	X			X	At5g05000	Toc34		X			X
At2g44640	LptD	X	X			X	At5g08540	Unknown	X	X		X	
*At3g01500*	CA1	X	X			X	At5g24650	Unknown		X		X	
*At3g04340*	emb2458	X			X		*At5g50920*	HSP93-V	X			X	
*At3g16950*	LPD1	X			X		*At5g66190*	FNR1	X	X			X
At3g17970	Toc64-III		X	X			*AtCg00480*	ATPB, PB	X	X			X
*At3g23400*	FIB4	X	X										

*Given is the Arabidopsis Genome Initiative (AGI) number (italic indicates category II), the short name and aliases, the identification in *A. thaliana* mixed envelope, *M. sativa* mixed envelope, *P. sativum* outer envelope, inner envelope, or mixed envelope. Identification of the protein is marked by an X in the column*.

The set of proteins found in all three species include amongst others solute transporters like LptD and Iep37 and as part of the IE/OE preprotein translocases Toc75-III, Toc159, and Tic55-II. Remarkably, only a single protein with unknown function was identified in all envelope fractions, namely At5g08540. Additionally, seven proteins of category II are detected in all species including the photosynthesis proteins LHCB6, PSAD-2, and ATPB. Furthermore, three proteins involved in signaling and response (CA1, RCA, and FNR1) and SDX1 of the lipid biosynthesis are identified. Remarkably, we could identify only one protein of category I in the envelope fractions of *A. thaliana* and *M. sativa*, which is the dually targeted (mitochondria and chloroplast) *S*-adenosylmethionine carrier 1 (SamC1; Palmieri et al., 2006). The category I proteins involved in transport (Oep16, NAP8), preprotein import (CJD1, Tic110), and signaling (MDH) could be detected in the envelope fractions of *A. thaliana* and *P. sativum* (Table 2). It appears that subfractionation of IE and OE membranes in case of the samples from *P. sativum* lead to an increased detection of preprotein import (Toc120, Tic55-IV, and Tic40) and transporter (Oep37, NAP14, MEX1, KEA2, DiT1, and DiT2.1) proteins. For the envelope fractions of *A. thaliana* and *M. sativa* only the preprotein import protein Toc75-V (*M. sativa*) and the transport proteins KEA1, TIP1.1, PIP2A, and PCaP1 in *A. thaliana* and Oep16-2 in *M. sativa* could be identified.

### The outer and inner envelope membrane proteome

**Figure 3:**
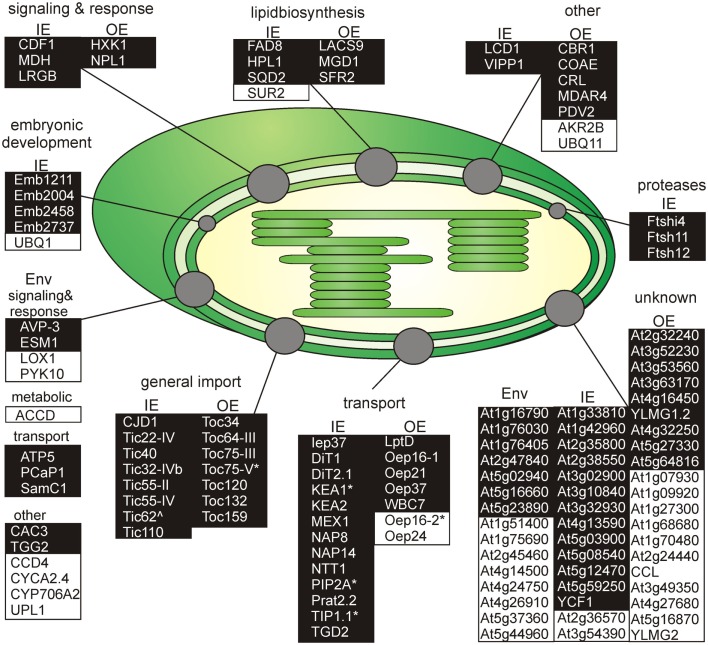
**Functional categorization of detected outer or inner envelope proteins**. Shown are all proteins clustered by their known functions (Tables 3– 5). The proteins of each cluster are sorted by the localization in the inner (IE) or in the outer envelope (OE) fraction. Proteins with an unclear localization (ENV) are listed in a separated cluster. Also proteins with unknown function (Table 6) are listed in a separated cluster. Black boxes indicate proteins of category I and white boxes proteins of category III (Table 1). * Indicates not found in ENV but clearly localized to IE or OE known from literature. ^∧^Indicates localized to IE or OE but only detected with one peptide.

Next, we inspected the individual proteomes of the OE and IE membrane of *P. sativum*, respectively. We only assigned proteins of categories I and III, which have been identified by at least two peptides. Due to the high uncertainty, proteins of category II were omitted (see above). Taking these criteria into account we could assign 30 proteins of known function to the OE (Table 3), and 34 proteins to the IE membrane (Table 4) and additional 22 proteins could not be clearly assigned (Table 5). In addition, we assigned 50 proteins of unknown function, 15 of them to the IE, 20 to the OE (fraction), and 15 to the envelope in general (Table 6). Thus, in total we were able to clearly assign 50 OE and 49 IE proteins (Figure 3) and will explain them in detail in the following sections.

**Table 3:** **Outer envelope proteins with known function**.

	AGI	Abbr.	Name and function	TM fold	Other Loc.	Studies	Cat.
**Preprotein import**	At2g16640	Toc132	GTP-binding chloroplast preprotein receptor	Unknown	–	3	I
	At3g16620	Toc120	GTP-binding chloroplast preprotein receptor	Unknown	n.d.	2	I
	At3g17970	Toc64-III	Chloroplast preprotein receptor	α-Helical TM	–	3	I
	At3g46740	Toc75-III	Translocon channel	β-Barrel	–	6	I
	At4g02510	Toc159	GTP-binding chloroplast preprotein receptor	Unknown	–	6	I
	At5g05000	Toc34	GTP-binding chloroplast preprotein receptor	1 α-Helical TM	–	6	I

**Lipid biosyn**.	At1g77590	LACS9	Long-chain acyl-CoA synthetase	1 α-Helical TM	–	6	I
	At3g06510	SFR2	Beta-glucosidase	2 α-Helical TM	–	6	I
	At4g31780	MGD1	Type A monogalactosyldiacylglycerol synthase	None	–	5	I

**Transport**	At1g20816	Oep21	Outer envelope channel	None	n.d./–	3	I
	At1g45170	Oep24	Outer envelope channel	None	–/n.d.	1	III
	At2g01320	WBC7	Putative subfamily G ABC-type transporter	4 α-Helical TM	–	3	I
	At2g28900	Oep16-I	Outer envelope protein	2 α-Helical TM	–	6	I
	At2g43950	Oep37	Outer membrane ion channel	β-Barrel	–	6	I
	At2g44640	LptD	Lipopolysaccharide-assembly protein D	β-Barrel	–	6	I

**Others**	At2g16070	PDV2	Plastid division machinery	1 α-Helical TM	–	3	I
	At2g17390	AKR2B	AKR2-like protein	None	n.d./–	1	III
	At2g27490	COAE	Putative dephospho-CoA kinase	None	X	2	I
	At3g27820	MDAR4	Membrane-associated monodehydroascorbate reductase	2 α-Helical TM	X	2	I
	At4g05050	UBQ11	Polyubiquitin	None	n.d.	1	III
	At4g29130	HXK1	Glucose-responsive sensor hexokinase	1 α-Helical TM	X	4	I
	At5g17770	CBR1	NADH:cytochrome b5 reductase	1 α-Helical TM	n.d./X	2	I
	At5g51020	CRL	Affects pattern of plastid division	1 α-Helical TM	–	2	I
	At5g58140	NPL1	Multifunctional blue-light-responsive photoreceptor	None	X	2	I

**Other Organelles**	At1g27390	Tom20-2	Putative mitochondrial outer membrane translocase component	1 α-Helical TM	n.d./X	3	I
	At3g46030	HTB11	Putative H2B-type histone	None	X/n.d.	1	III
	At4g14430	ECHIb	Putative enoyl-CoA hydratase/isomerase	1 α-Helical TM	X	1	III
	At4g35000	APX3	Putative peroxisomal ascorbate peroxidase	1 α-Helical TM	X	5	I
	At4g38920	VHA-C3	c-Type subunit of vacuolar H(+)-ATPase membrane V0 subcomplex	4 α-Helical TM	n.d./X	1	III
	At5g43070	WPP1	Nuclear envelope-targeted protein involved in mitotic activity	None	n.d./X	2	I

*Given is the functional pathway or the organellar compartment, the AGI number, the short name (Abbr.), the (putative) function, the transmembrane anchor architecture, other localization by the PPDB (Sun et al., 2009) and SUBAII (Heazlewood et al., 2007; n.d. not defined, –, no other localization, X, other localization), the number of studies where the protein was identified (our study; Ferro et al., 2003; Froehlich et al., 2003; Bräutigam et al., 2008; Bräutigam and Weber, 2009; Ferro et al., 2010), and the category from our study. Lipid biosynthesis (Lipid biosyn.)*.

**Table 4:** **Inner envelope proteins with known function**.

	AGI	Abbr.	Name and function	TM fold	Other Loc.	Studies	Cat
**Preprotein import**	At1g06950	Tic110	Inner envelope translocon component	1 α-Helical TM	–	6	I
	At1g08640	CJD1	DnaJ-like membrane protein	3 α-Helical TM	–	5	I
	At2g24820	Tic55-II	Inner envelope Rieske iron-sulfur protein	3 α-Helical TM	–	5	I
	At4g23420	Tic32-IVb	NAD- or NADP-dependent oxidoreductase	1 α-Helical TM	n.d.	2	I
	At4g25650	Tic55-IV	Inner envelope Rieske iron-sulfur protein	2 α-Helical TM	–	4	I
	At4g33350	Tic22-IV	Inner envelope translocon component	None	–	6	I
	At5g16620	Tic40	Inner envelope translocon component	1 α-Helical TM	–	6	I

**Lipid biosyn**.	At4g15440	HPL1	Membrane-associated hydroperoxide lyase	2 α-Helical TM	–	4	I
	At4g31500	SUR2	Cytochrome P450 monooxygenase	2 α-Helical TM	X	1	III
	At5g01220	SQD2	UDP-sulfoquinovose:DAG sulfoquinovosyltransferase	1 α-Helical TM	–	4	I
	At5g05580	FAD8	chloroplast omega-3 fatty acid desaturase	3 α-Helical TM	–	3	I

**Transport**	At1g80300	NTT1	Plastidic ATP/ADP antiporter	11 α-Helical TM	–	5	I
	At3g20320	TGD2	Putative subfamily I ABC protein	1 α-Helical TM	–	6	I
	At3g63410	IEP37	37 kDa chloroplast inner envelope protein	2 α-Helical TM	–	6	I
	At4g00630	KEA2	Putative potassium cation efflux antiporter	14 α-Helical TM	–	4	I
	At4g25450	NAP8	Putative subfamily B ABC-type transporter	5 α-Helical TM	–	6	I
	At5g12860	DiT1	Plastidic 2-oxoglutarate/malate-translocator	13 α-Helical TM	–	6	I
	At5g14100	NAP14	Putative subfamily I ABC protein	None	–	3	I
	At5g17520	MEX1	Putative maltose translocator	9 α-Helical TM	–	3	I
	At5g24650	PRAT2.2	Putative dual-targeted mitochondrial and plastidial membrane translocase	4 α-Helical TM	–	6	I
	At5g64290	DiT2.1	Plastidic glutamate/malate-translocator	11 α-Helical TM	–	6	I

**SR**	At1g32080	LRGB	LrgB-like membrane protein	12 α-Helical TM	–	5	I
	At3g47520	MDH	NAD-malate dehydrogenase	1 α-Helical TM	–	4	I
	At5g23040	CDF1	Cell growth defect factor	3 α-Helical TM	–	5	I

**Proteases**	At1g79560	FtsH12	ATP-dependent metalloprotease	2 α-Helical TM	–	4	I
	At5g53170	FtsH11	ATP-dependent metalloprotease	1 α-Helical TM	X	4	I
	At5g64580	FtsHi4	Putative ATP-dependent metalloprotease	1 α-Helical TM	–	4	I

**Embryon. develop**.	At1g10510	Emb2004	Putative membrane protein	1 α-Helical TM	–	6	I
	At3g04340	Emb2458	Putative membrane protein	3 α-Helical TM	–	4	I
	At3g52590	UBQ1	Ubiquitin extension protein	None	X	1	III
	At5g22640	Emb1211	Putative membrane protein	None	–	4	I
	At5g53860	Emb2737	Putative membrane protein	None	–	3	I

**Others**	At1g65260	VIPP1	Membrane-associated vesicle-inducing prot.	None	–	6	I
	At2g37860	LCD1	Mutant lcd1-1 exhibits pale phenotype	2 α-Helical TM	–	4	I

*Given is the functional pathway or the organellar compartment, the AGI number, the short name (Abbr.), the (putative) function, the transmembrane anchor architecture, other localization by the PPDB (Sun et al., 2009) and SUBAII (Heazlewood et al., 2007; n.d., not defined, –, no other localization, X, other localization), the number of studies where the protein was identified (our study; Ferro et al., 2003; Froehlich et al., 2003; Bräutigam et al., 2008; Bräutigam and Weber, 2009; Ferro et al., 2010), and the category from our study. Lipid biosynthesis (Lipid biosyn.); signaling and response (SR); embryonic development (Embryon. develop.)*.

**Table 5:** **Mixed envelope proteins with known function**.

	AGI	Abbr.	Name and function	TM fold	Other Loc.	Studies	Cat.
**Preprotein import**	At5g19620	Toc75-V	Protein translocation channel at OEM	β-Barrel	–	5	I

**Lipid biosyn**.	AtCg00500	ACCD	Carboxyltransferase beta-subunit of acetyl-CoA carboxylase complex	None	–	1	III

**Transport**	At1g01790	KEA1	Putative potassium cation efflux antiporter	13 α-Helical TM	–	6	I
	At2g36830	TIP1.1	Putative tonoplast intrinsic protein	6 α-Helical TM	n.d./–	3	I
	At3g53420	PIP2A	Putative plasma membrane intrinsic protein 2a	6 α-Helical TM	–	2	I
	At4g16160	Oep16-2	Putative plastid outer envelope protein	β-Barrel	–	1	III
	At4g20260	PCaP1	Mediates hypocotyls cell elongation	None	–	2	I
	At4g39460	SamC1	*S*-Adenosylmethionine transporter	5 α-Helical TM	–	5	I
	At5g13450	ATP5	Mitochondrion MF1-ATP synthase subunit	None	X	3	I

**SR**	At1g15690	AVP-3	Type I proton-translocating pyrophosphatase	14 α-Helical TM	–	4	I
	At1g55020	LOX1	Lipooxygenase	1 α-Helical TM	n.d.	1	III
	At3g09260	PYK10	Beta-glucosidase	1 α-Helical TM	X	1	III
	At3g14210	ESM1	Putative GDSL-type lipase	1 α-Helical TM	n.d./–	2	I

**Others**	At1g55860	UPL1	Putative ubiquitin-protein ligase 1	1 α-Helical TM	n.d./X	1	III
	At1g80370	CYCA2_4	A-type cyclin	1 α-Helical TM	n.d./X	1	III
	At2g38040	CAC3	Alpha subunit of acetyl-CoA carboxylase complex	None	–	6	I
	At4g19170	CCD4	Putative carotenoid cleavage dioxygenase	None	–	1	III
	At4g22710	CYP706A2	Cytochrome P450 monooxygenase	1 α-Helical TM	n.d./–	1	III
	At5g25980	TGG2	Thioglucoside glucohydrolase	1 α-Helical TM	n.d./–	2	I

**Other Organelle**	At1g78900	VHA-A	A-type subunit of vacuolar H(+)-ATPase peripheral V1 subcomplex	None	n.d./–	4	I
	At2g38670	PECT1	Phosphoethanolamine cytidylyltransferase	1 α-helical TM	n.d./X	2	I
	At5g15920	SMC5	Putative SMC5-like component of chromosome metabolism	None	n.d.	1	III

*Given is the functional pathway or the organellar compartment, the AGI number, the short name (Abbr.), the (putative) function, the transmembrane anchor architecture, other localization by the PPDB (Sun et al., 2009) and SUBAII (Heazlewood et al., 2007; n.d., not defined, –, no other localization, X, other localization), the number of studies where the protein was identified (our study; Ferro et al., 2003; Froehlich et al., 2003; Bräutigam et al., 2008; Bräutigam and Weber, 2009; Ferro et al., 2010), and the category from our study. Signaling and response (SR)*.

**Table 6:** **Proteins from Category I and III with unknown function**.

Loc.	AGI	Putative function^A^ or Closest homologue^B^ or GO annotation^C^	Putative TM fold	Studies	Cat.
**Inner envelope membrane**	At1g33810	TIM phosphate binding super family^A^	1 α-Helical TM	5	I
	At1g42960	Glutaredoxin2 C^A^	1 α-Helical TM	6	I
	At2g35800	Mitochondrial glutamate carrier (*M. truncatula*)^B^	2 α-Helical TM	4	I
	At2g36570	Leucine-rich repeat receptor-like proteinkinase (Tyrosin kinase)^A,B^	1 α-Helical TM	1	III
	At2g38550	Transmembrane proteins 14C (*H. sapiens*)^B^	4 α-Helical TM	6	I
	At3g02900	YCF1.2 protein^A^	1 α-Helical TM	4	I
	At3g10840	Putative alpha/beta-fold-type hydrolase^A,B^	2 α-Helical TM	4	I
	At3g32930	Pterin 4 alpha carbinolamine dehydratase^A^	None	4	I
	At3g54390	Putative DNA-binding protein (SWI3, ADA2, N-CoR, and TFIIIB)^A^	None	1	III
	At4g13590	PF27 (small family from bacteria and eukaryotes) belongs to the lysine exporter superfamily)^A^	7 α-Helical TM	4	I
	At5g03900	Iron-sulfur cluster biosynthesis family protein^A^	2 α-Helical TM	4	I
	At5g08540	Oligonucleotide/oligosaccharide binding DNA ligase family^A^	1 α-Helical TM	6	I
	At5g12470	DUF3411 and Glycine rich proteien family^A^	4 α-Helical TM	6	I
	At5g59250	Putative sugar transporter^A/^D-xylose-proton symporter-like protein^B^	11 α-Helical TM	6	I
	AtCg01130	Ycf1 protein^A/^Ycf1 (*B. napus*)^B^	8 α-Helical TM	3	I

**Outer envelope membrane**	At1g07930	Putative elongation factor Tu GTP-binding protein^A,B^	None	1	III
	At1g09920	Putative PRLI-interacting factor K^A^	1 α-Helical TM	1	III
	At1g27300	Protein binding^C^	1 α-Helical TM	1	III
	At1g68680	Putative mitochondrial respiratory chain complex I^C^	2 α-Helical TM	1	III
	At1g70480	Putative steroidogenic acute regulatory protein; lipid transfer protein^A^	None	1	III
	At2g24440	Putative protein that binds to UDP-glucose:glycoprotein glucosyltransferase^A^/selT/selW/selH selenoprotein (*Z. mays*)^B^	None	1	III
	At2g32240	Putative SMC protein that bind DNA and act in organizing and segregating chromosomes for partition^A^	1 α-Helical TM	2	I
	At3g26740	CCL putative light regulated protein^A,B^	None	1	III
	At3g49350	Putative GTPase activator protein of Rab-like small GTPases^A,B^	None	1	III
	At3g52230	Chloroplast outer envelope 24 kD protein like (omp24)^B^	1 α-Helical TM	6	I
	At3g53560	Putative protein protein interaction function^A^	None	2	I
	At3g63170	Putative chalcone-flavonone isomerase^A,B^	1 α-Helical TM	4	I
	At4g16450	Putative NADH-ubiquinone oxidoreductase complex I^A,B^	1 α-Helical TM	3	I
	At4g27680	Putative ATPases associated ^A/^Spastin (*M. truncatula*)^B^	1 α-Helical TM	1	III
	At4g27990	YLMG1-2 (YGGT family protein) YGGT repeat found in conserved hypothetical integral membrane proteins^A^	3 α-Helical TM	5	I
	At4g32250	Putative Serine/Theronine protein kinase^A/^G protein-coupled receptor kinase (*M. truncatula*)^B^	1 α-Helical TM	3	I
	At5g16870	Putative peptidyl-tRNA hydrolase (PTH2)^A,B^	1 α-Helical TM	1	III
	At5g21920	YLMG2 (YGGT family protein) YGGT repeat found in conserved hypothetical integral membrane proteins^A^	2 α-Helical TM	1	III
	At5g27330	Putative SMC protein that bind DNA and act in organizing and segregating chromosomes for partition^A^	1 α-Helical TM	2	I
	At5g64816	Not determined	1 α-Helical TM	3	I

**Mixed envelope membrane**	At1g16790	Ribosomal protein related^C^	None	5	I
	At1g51400	Photosystem II reaction center subunit T^A,B^	None	1	III
	At1g75690	Putative chaperone protein dnaJ-related^A^	1 α-Helical TM	1	III
	At1g76030	V-type (H +)-ATPase V1 like protein^A,B^	None	2	I
	At1g76405	Putative Cupin-like protein^A^/Outer envelope pore protein of 21 kDa (*P. sativum*)	None	3	I
	At2g45460	FHA Putative nuclear signaling domain (FHA)^A^	None	1	III
	At2g47840	Chloroplast protein import component Tic20/Ycf60 famly protein^A,B^	3 α-Helical TM	6	I
	At4g14500	Putative steroidogenic acute regulatory lipid transfer protein^A,B^	3 α-Helical TM	1	III
	At4g24750	Rhodanese homology domain^A^	1 α-Helical TM	1	III
	At4g26910	Putative 2-oxoglutarate dehydrogenase E2 component^A,B^	None	1	III
	At5g02940	DUF1012^A/^Os03g163100 (*O. sativa*)^B^	3 α-Helical TM	4	I
	At5g16660	Apolipoprotein domain^A/^Os12g0583400 (*O. sativa*)^B^	1 α-Helical TM	4	I
	At5g23890	Os03g0862100 (*O. sativa*)^B^	1 α-Helical TM	6	I
	At5g37360	Ammonium transporter AES61175.1 (*M. truncatula*)^B^	2 α-Helical TM	1	III
	At5g44960	Putative F-box like protein associated in nuclear processes^A^	None	1	III

*Given is the fraction the protein was localized in (outer/inner/mixed envelope membrane), the AGI number, putative function or functional domain indicated by ^A^ or closest homolog indicated by ^B^ or function annotated in GeneOntology (GO) ^C^, the transmembrane architecture, the number of studies where the protein was identified with (our study; Ferro et al., 2003; Froehlich et al., 2003; Bräutigam et al., 2008; Bräutigam and Weber, 2009; Ferro et al., 2010), and the category*.

#### Outer envelope proteins

We identified homologs to known OE proteins such as components of the TOC complex (Schleiff and Becker, 2011), like Toc75-III, Toc34, Toc159, Toc120 and Toc132, and Toc64-III which have been previously reported (Schleiff et al., 2003a; Ladig et al., 2011). The latter three were exclusively found in the OE membrane. Remarkably, we were not able to detect Toc75-V, except in the envelope fraction of *M. sativa* (Table 5). Further identified proteins with confirmed OE localization were Oep37, Oep21, and Oep16 (Schleiff et al., 2003a), SENSITIVE TO FREEZING 2 protein (Sfr2), a galactolipid-remodeling enzyme (Fourrier et al., 2008; Moellering et al., 2010) and CRUMPLED LEAF protein (Crl) and PDV2, which are both involved in plastid division (Asano et al., 2004; Glynn et al., 2008).

Additionally, we included proteins, for which significantly more peptides were found in the OE than in the IE fraction, albeit their exact localization is unclear. The long-chain acyl-CoA synthetase Lacs9 (Schnurr et al., 2002), the ABC-type transporter WBC7 (Ferro et al., 2003), and the paralog of TGD4 (Xu et al., 2008) encoded by AT2G44640 (LptD; Haarmann et al., 2010) were shown to be localized in the envelope membranes, before (Ferro et al., 2003; Froehlich et al., 2003). Similarly, the kinase CoaE was identified in the chloroplast proteome, but experimental data on the localization does not exist (Zybailov et al., 2008).

The protein encoded by AT5G27330 is annotated as Prefold in chaperone subunit family protein and was predicted to be localized in the endoplasmic reticulum (Dunkley et al., 2006). Likewise, ascorbate peroxidase Apx3 (Narendra et al., 2006) was previously assigned to peroxisomal membranes, while Cbr1 (Fukuchi-Mizutani et al., 1999) was described as a protein of the microsomal electron-transfer system. Remarkably, both proteins were identified as substrates of the Akr2a-dependent transport (Shen et al., 2010), which is also involved in the transport of Oep7 to the chloroplast OE membrane (Bae et al., 2008). Furthermore, Apx3 was previously identified in the chloroplast proteome (Zybailov et al., 2008). Although unclear, these proteins are most likely dually localized to both, peroxisomes or ER and chloroplasts.

In contrast, we identified a couple of proteins, which indicate a slight impurity of the sample, namely Mdar4 (Lisenbee et al., 2005), which was clearly assigned to the peroxisomal membrane, Wpp1 (Patel et al., 2004) and Hxk1 (Moore et al., 2003), which are nuclear proteins, the mitochondrial proteins Tom20 and AT4G16450 (Lister et al., 2007; Klodmann et al., 2010) vacuolar protein AVA-P3, and IE protein MGD1, the MGDG synthase (Awai et al., 2001; Ladig et al., 2011). Further, LHCB1.4 is a thylakoid protein. AT4G27680, AT3G52230, AT2G32240, AT3G53560, AT2G24440, AT1G09920, AT3G49350, and AT1G68680 are unknown, while for the protein kinase encoded by AT4G32250 a stromal localization was proposed (Friso et al., 2004; Zybailov et al., 2009).

#### Inner envelope proteins

Analyzing the IE proteome of *P. sativum*, we realized that it was in contrast to the OE fraction heavily contaminated with proteins of the stroma and the OE. First, with clearly annotated OE proteins like Toc75-III, Toc159, Toc34, Lacs9, and Oep21. Second, with stromal proteins like the small subunit of ribulose bisphosphate carboxylase Rbcs1A, Rbcl and the ATP-dependent RuBisCO activase (RCA), the malate dehydrogenase (MDH), and subunit PsaG of photosystem I complex as prominent stromal contaminations. For Emb1211, PsaD-2, the beta-subunit of ATP synthase (ATPB), AT1G33810, and the geranyl reductase AT1G74470 a thylakoid localization was determined (Peltier et al., 2004).

As expected, we identified proteins of the preprotein translocon of the inner membrane (TIC; Soll and Schleiff, 2004), namely Tic110, Tic55-II, Tic55-IV, Tic40, and Tic32-IVb as major components of the IE fraction. Although assigned to category II we identified IE membrane-associated cpHsp70 (two peptides; Su and Li, 2010) and CPN60 (two peptides; Stürzenbaum et al., 2005), which were two chaperones previously discussed to be involved in preprotein import. We also detected two peptides for intermembrane space localized Tic22-IV. Remarkably, the chloroplast-targeted ferredoxin-NADP(+)-oxidoreductase FNR1 (Table 4), which was found to be associated with the IE via interaction with Tic62 before (Küchler et al., 2002), was clearly detected, whereas Tic62 was identified by only one peptide. Similarly, for Tic20 we found only a single peptide as well. The absence or the low coverage of the membrane-inserted TIC proteins might reflect the problems of analyzing membrane proteins in general (Eichacker et al., 2004).

Besides the TIC components, we identified Iep37, which is described as an IE protein involved in Polyquinone biosynthesis (Dreses-Werringloer et al., 1991). Similarly, the cell growth defect factor Cdf1 (Kawai-Yamada et al., 2005), which is able to induce apoptosis when expressed in yeast, was found to be localized in the IE of chloroplasts (Ladig et al., 2011). Sulfoquinovosyldiacylglycerol (SQDG) synthesis occurs in envelope membranes (Seifert and Heinz, 1992) and here identified SQDG synthase (SQD2; Yu et al., 2002) was localized in chloroplasts. Similarly, we detected the stromal FAD8 (Matsuda et al., 2005) involved in lipid desaturation, and TGD2 involved in transport of lipids from the ER to chloroplasts (three peptides; Awai et al., 2006).

Further, we detected the ATP/ADP antiporter of the IE (NTT1; Neuhaus et al., 1997), the preprotein and amino acid transporter family protein Prat2.2 (Murcha et al., 2007) and the potassium cation efflux antiporter KEA2 (Zybailov et al., 2008) with at least four peptides. In addition, one peptide each was found for the putative magnesium cation transporter MGT10 (Froehlich et al., 2003), for the triose-phosphate/phosphate translocator TPT (Schneider et al., 2002), for the mitoferrin-like carrier MFL1 (Tarantino et al., 2011), for the plastidial sodium-dependent pyruvate transporter BAT1 (Furumoto et al., 2011), and two peptides for the plastidic glutamate/malate-translocator (DIT2; Renné et al., 2003), the putative sugar transporter encoded by AT5G59250 (Froehlich et al., 2003), as well as three peptides for the plastidic 2-oxoglutarate/malate-translocator (DIT1; Weber et al., 1995), and for the maltose transporter Mex1 (Niittylä et al., 2004).

The beta-carbonic anhydrase (CA1; Fabre et al., 2007) of category II and the three metalloproteases (category I; FtsH4i, FtsH11, and FtsH12), detected in the IE fraction, were previously allocated to the stroma (Sakamoto et al., 2003), but they might be associated with the IE as well as suggested for Emb2458 (Froehlich et al., 2003). The same holds true for the DnaJ-like membrane protein of unknown function (CJD1; Zybailov et al., 2008). The tocopherol cyclase SXD1 (category II; Provencher et al., 2001) is chloroplast-localized and is involved in tocopherol synthesis, which takes place in the IE membrane (Lichtenthaler et al., 1981). Thus, it is most likely that these six proteins are membrane-associated and correctly assigned to the IE membrane. The proteins encoded by AT1G33810, AT1G42960, AT2G35800, AT2G38550, AT3G02900, AT3G10840, AT3G32930, AT4G13590, AT5G03900, AT5G08540, and AT5G12470 are assigned as (inner) envelope proteins (Ferro et al., 2003, 2010; Froehlich et al., 2003; Bräutigam et al., 2008; Bräutigam and Weber, 2009), but their function remains to be explored. We further confirmed the IE localization of the plastid-encoded Ycf1.2 (Ladig et al., 2011). The latter might be inserted by the recently identified Sec translocon (Skalitzky et al., 2011).

#### Non-assignable and unknown proteins

Next to the proteins with known functions that could be clearly assigned to the OE/IE membrane in *P. sativum*, we identified two additional classes of proteins. The first are proteins that have a known function but could not clearly be allocated to either of the membranes (Table 5), because these proteins were found only in the mixed envelope of *A. thaliana* and/or *M. sativa*. Most of these proteins function as transporters like KEA1, TIP1.1, PIP2A, Oep16-2, PCaP1, and SamC1 or signaling and response (AVP-3, LOX1, PYK10, and ESM1). Toc75-V (Schleiff et al., 2003a) was the only preprotein import protein, which could be identified in the mixed envelope fraction but not in the OE or IE membrane of *P. sativum*.

**Figure A2:**
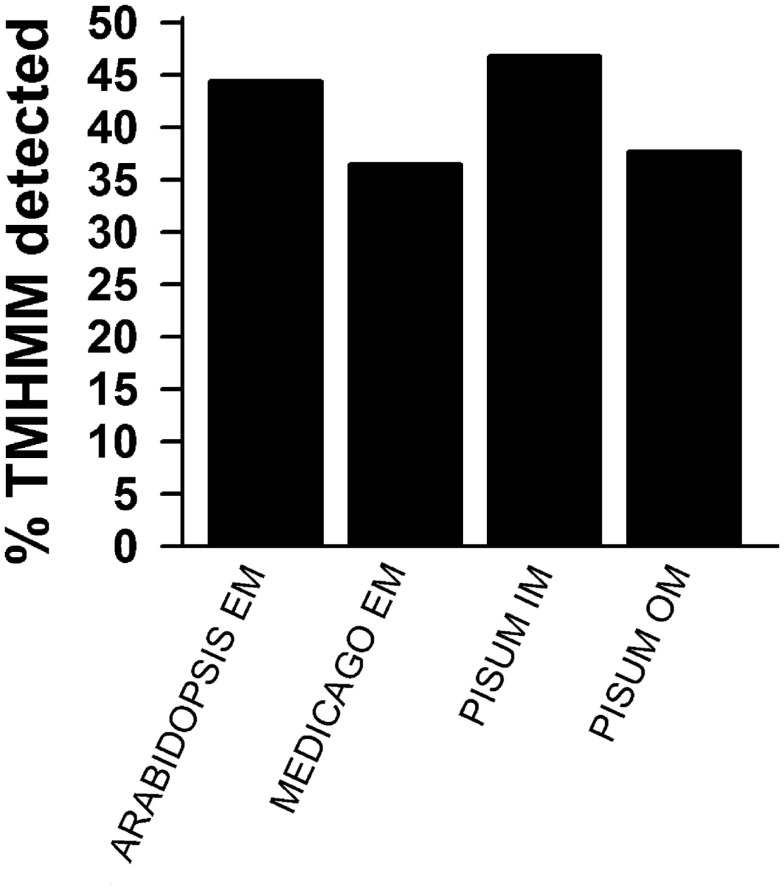
**Prediction of membrane localization**. TOPCONS single was used to predict transmembrane helices. The percentage of identified proteins for the different pools as described in Figures 1D,E is presented. Shown are the results for all proteins identified by more than one peptide (black) and for all identified proteins.

The second are proteins of which neither function nor localizations are known yet (Table 6). These proteins were assigned concerning their identification in OE or IE membrane of *P. sativum*. Two of 15 IE-assigned proteins of unknown function (At2g36570, At3g54390) were only detected in our study, whereas ∼50% of OE-assigned unknown proteins are of category III. To characterize the unknown proteins of the two groups and support them as potential new IE/OE envelope proteins we used TOPCONS single (Figure A2 in Appendix, Hennerdal and Elofsson, 2011) and Aramemnon (Schwacke et al., 2003) for secondary structure prediction. Eighty-five percent of the unknown IE proteins possess at least one predicted transmembrane helix (Table 6) and might therefore be anchored or embedded into the IE membrane. None of the unknown OE proteins are found to be β-barrel structures, which would have been an argument for an OE localization (Schleiff et al., 2003a) However, it has to be taken into account, the prediction of eukaryotic β-barrel proteins is not as reliable as of helical proteins (Mirus and Schleiff, 2005). Also, the putative function via Pfam (Finn et al., 2010) and CDD (Marchler-Bauer and Bryant, 2004) and the closest homolog via reciprocal best BLAST hit search were predicted to allocate the proteins correctly (Table 6). Interestingly, most of the unknown proteins assigned to the IE are localized via PPDB and SUBAII to the plastid except of At2g36570 (other localization) and At3g54390 (not determined), whereas most of the OE-assigned proteins are not determined at least by one database and only six proteins are localized in the plastid (At3g26740, At3g52230, At3g53560, At3g63170, At4g27990, and At4g32250).

## Experimental Section

### Isolation and fractionation of chloroplasts

#### Arabidopsis thaliana

Chloroplasts were isolated from 20-day-old *A. thaliana* plants (Col-0 ecotype Columbia; 8 h light/16 h dark photoperiod of 120 μmol m^−2^ s^−1^; 25°C). Plants were harvested before light onset and all procedures were carried out at 4°C. Leaves were cut and homogenized in 450 mM Sorbitol, 20 mM Tricin-KOH pH 8.4, 10 mM EDTA, 5 mM NaHCO_3_, 1 mM PMSF, using a waring blender (four pulses: low speed 3 s; medium speed 3 s; high speed 2 s; low speed 4 s). The homogenate was filtered through four layers of cheesecloth and one layer of miracloth and centrifuged for 5 min at 1,500 × *g* and 4°C. The pellet was resuspended using a paintbrush in 300 mM Sorbitol, 20 mM Tricin-KOH pH 7.6, 5 mM MgCl_2_, 2.5 mM EDTA, 1 mM PMSF (resuspension buffer), placed on top of percoll gradients by underlying 12 ml of 45% (v/v) Percoll™ with 8 ml of 85% (v/v) Percoll™, and centrifuged for 10 min at 10,000 × *g*. Intact chloroplasts between 40 and 80% (v/v) Percoll™ were collected after removal of broken chloroplasts by water jet pump. Intact chloroplasts were washed twice by centrifugation for 5 min at 1,500 × *g* in resuspension buffer and collected.

Chloroplasts were lysed by resuspension in 10 mM Tris-HCl pH 8.0, 1 mM EDTA, 1 mM PMSF (TE buffer) to a final concentration of 2 mg chlorophyll/ml. The suspension was placed on top of a sucrose step-gradient (2.4 ml 1.2 M; 4 ml 1.0 M; 4 ml 0.45 M sucrose in TE buffer) and centrifuged for 2 h at 125,000 × *g* and 4°C. Chloroplast fractions were recovered by Pasteur pipettes, diluted 1:3 in TE buffer, centrifuged, pooled, and immediately frozen in liquid nitrogen and stored in −80°C.

#### Pisum sativum

Chloroplast isolation was adapted from Schleiff et al. (2003a,b). Pea (*P. sativum* cv. Arvika) plants were grown for 8 days in a greenhouse (8 h dark/16 h light, 70 μmol m^−2^ s^−1^; 25°C). Pea leaves were harvested and homogenized in the 330 mM Sorbitol, 13 mM Tris, 20 mM MOPS, 0.1 mM MgCl_2_, 0.02% (w/v) BSA, 1 mM β-ME, 0.3 mM PMSF using a waring blender (five pulses, low/medium/high/low/medium, all 2 s). The suspension was filtered through four layers of cheesecloth and one layer of miracloth and centrifuged for 5 min at 1,500 × *g* and 4°C. The pellet was resuspended in the remaining buffer, transferred with cut 5 ml-pipette tip on top of Percoll gradients prepared by underlaying 13 ml of 40% (v/v) Percoll™ with 8 ml of 80% (v/v) Percoll™, centrifuged for 10 min at 10,000 × *g* and 4°C. Intact chloroplasts were collected from the phase between 40 and 80% Percoll™ and washed twice in 330 mM Sorbitol, 1 mM β-ME, and 0.3 mM PMSF.

Chloroplasts were osmotically shocked by adding 2.4 M sucrose solution to a final concentration of 0.6 M sucrose and incubation for 10 min in dark, followed by mechanical disruption with 50 strokes in a dounce homogenizer. Solution was mixed with 2.4 M sucrose solution to a final concentration of 1.35 M, overlayed with 10 ml 1.1 M, 10 ml 1.0 M, and 8 ml 0.45 sucrose solutions, respectively. Chloroplast sub-compartments were recovered after centrifugation for 18 h at 125,000 × *g* and 4°C, resuspended 10 mM Tris-HCl pH 8.0, 1 mM EDTA, 1 mM PMSF, and stored in −80°C.

#### Medicago sativa

Chloroplast isolation, and subsequent fractionation, from Alfalfa seedlings was performed as described for pea chloroplast with the following modifications. Seedlings were grown for 20 days and leaves were homogenized in a waring blender (2 × 3 pulses at low speed for 3 s; at medium speed for 3 s at high speed for 2 s). Further, Percoll™ gradients were prepared by underlying 13 ml of 42% (v/v) Percoll™ with 8 ml of 82% (v/v) Percoll™.

### Proteome analysis by MALDI nano-LC-MS/MS

#### Preparation for enzymatic digestion

An amount of 120 μg membranes were washed using 25 mM NH_4_HCO_3_ pH 8.0 and carbamidomethylated prior to digestion. After 2 min of centrifugation at 12,000 × *g* the supernatant was removed, and the pellet was gently resuspended in 100% (v/v) methanol. Sample reduction with DTT was performed at 56°C for 45 min and 10 μl of a 500 mM iodoacetamide in 25 mM NH_4_HCO_3_ solution was used for sulfhydryl alkylation. Following a 10 min period of sonication, the methanol was diluted to 60% (v/v) using 25 mM NH_4_HCO_3_ buffer. The proteolytic digestion was performed by adding either 2 μg of trypsin (three biological replicates each organism and envelope fraction) or 10 μg of elastase (three biological replicates each organism and envelope fraction) for 16 h at 36°C. Prior to storing at −20°C the peptide-containing sample was centrifuged at 12,000 × *g* for 2 min in order to remove all undigested membranes and finally the supernatant was concentrated to 15 μl.

#### Mass spectrometry

Extracted peptides were subjected to MALDI nLC-MS/MS. Specifically, extracted peptides were injected into an Easy-nLC from Proxeon Systems (Thermo Fisher Scientific, Dreieich, Germany) using solvent A [8% (v/v) acetonitrile, 0.1% (v/v) trifluoric acid]. Separation was performed on a thermostatic (40°C) custom made C_18_ column (X-Bridge™ BEH 180 C_18_ 300 Å 3.5 μm, 75 μm × 150 mm) at a flow rate of 300 nl/min with increasing acetonitrile concentrations. The linear-gradient profile was used for tryptic peptide digests started with 8–90% solvent B [95% (v/v) acetonitrile, 0.1%(v/v) trifluoric acid] in 75 min, a stagnation at this level for 8 min, followed by a quick decline to 8% in 5 min and finally, an additional 2 min at 8% for column equilibration. In the case of elastase generated peptide mixtures, the linear-gradient profile duration was increased to 105 min. The separated peptides were then mixed on a tee (Upchurch Scientific) with matrix solution supplied by an auxiliary pump (flow rate, 1.0 μl/min). This solution contained 3 mg/ml α-Cyano-4-hydroxycinnamic acid (α-CHCA; Bruker Daltonics, Germany) dissolved in 70% (v/v) acetonitrile, 30% (v/v) H_2_O, and 0.1% (v/v) trifluoric acid. The final mixture was directly spotted every 20 s on a blank 123 mm × 81 mm Opti-TOF™ LC/MALDI insert metal target. Subsequent MALDI-TOF/TOF measurements were carried out using the 4800 TOF/TOF Analyzer (Applied Biosystems, Germany). All peptides used for calibration were taken from the Sequazyme™ Peptide Mass Standards kit (Applied Biosystems, Germany). Spectra were acquired in the positive reflector mode between 700 and 4000 *m*/*z* with fixed laser intensity. A total of 750 laser shots per spot were accumulated. The precursor selection for MS/MS was carried out via the software of the instrument to avoid unnecessary multiple selections of identical precursor peptides. Up to 10 precursors per spot were selected for fragmentation each requiring a minimum signal-to-noise ratio of 30. The fragmentation of the selected precursors was performed at collision energy of 1 kV using air as collision gas at a pressure of 1 × 10^-6^ torr. Depending on the spectral quality, 1250–2500 laser shots were recorded. Potential matrix cluster signals were removed from precursor selection by excluding all masses in the range from 700 to 1400 *m*/*z* having values of 0.030 ± 0.1 *m*/*z* as well as the internal calibrant mass.

### Data analysis and presentation

#### Format parsing

Mascot generic format (mgf) files were retrieved from each nLC-MALDI MS/MS run (three biological replicates each organism and envelope fraction; Table S14 in Supplementary Material) using the built-in Peaks2Mascot feature, exporting up to 65 peaks per MS/MS spectrum, each requiring a minimum signal-to-noise of 5. MS/MS queries were processed using the Mascot database search engine v2.2.03 (Matrix Science Ltd.). Data were analyzed using the following settings: below 60 ppm MS precursor mass tolerance (except for the OE of *P. sativum* in combination with trypsin which was 90 ppm due to a technical problem with the instrument that day) and below 0.5 Da MS/MS mass tolerance for MALDI-TOF/TOF. For all database searches, the post-translational modifications carbamidomethylation of cysteins and oxidation of methionines were both selected as variable. When tryptic searches were performed, up to three missed cleavages were taken into consideration in combination with a specific cleavage after K and R and not before P. In all elastase searches, the number of missed cleavages was set to the maximum value of 9 and enzyme specificity was set to A, V, L, I, S, and T, but not before P. For all samples, a custom Viridiplantae database was generated from UniProtKB containing 887,260 entries as of March 02, 2011. Additionally, for *P. sativum* and *M. sativa* samples, customized databases containing 79,106 and 47,532 sequences were provided by the EST-library (Franssen et al., 2012) and MT3.0 of the IMGAG^1^, respectively. False discovery rates (FDR, Table S14 in Supplementary Material) given are those originating from the internal Mascot decoy database search function. For each nLC-MALDI-MS/MS run and each sample, the ions score cut off was calculated individually as −10 log (*p*) with *p* = 0.05 (95% confidence level; Table S14 in Supplementary Material). The Mascot analyses were described in the paper of Rietschel et al. (2009). For multiple fragmentations of identical precursors, due to the reappearance in repetitions, only data from the highest scoring peptide were kept. Significant proteins present in all three triplicates were taken and summarized in one table for each type of experiment. Afterward, these tables of elastase and trypsin treatments, containing non-identical hits and peptides, were fused.

#### Peptide assignment

Depending on the source the peptides identified by Mascot or Sequest were afterward aligned either to the protein database of TAIR9 (*A. thaliana*^2^), the protein database of MIPS (*M. truncatula*^3^), or the data file of contigs und singlets (*P. sativum*, data file from Franssen et al. (2012) using a standalone version of Blast from NCBI (substitution matrix BLOSUM62 with linear gap penalty). Following criteria were applied: peptides were only assigned to proteins in the database, if (i) they were aligned with an identity of >95% (determined via blastp), (ii) they had no gaps or mismatches except for (iii) a single substitution with amino acid residues with similar qualities (defined by the substitution matrix) or a single undefined amino acid position (declared by X). Short peptides (<11 aa), which were already covered by assigned peptides, were not subject to the previously mentioned criteria. Those short peptides were assigned to the protein, although they were not aligned with BLAST, which is insufficiently accurate regarding the assignment of peptides shorter than 11 amino acids. This method was used to reduce redundancy and as a more stringent criterion for the detection of proteins via the predicted peptides of Mascot. Also, we used a single method to assign the different species and databases in the same way under the same parameter settings of BLAST. Additionally, we searched in parallel for the closest homolog of *A. thaliana* in the other species.

The peptides allocated to *P. sativum* or *M. truncatula* are also allocated to the possible orthologs in *A. thaliana*. On the basis of the *A. thaliana* gene identifiers and their allocated peptides, the splice variants of the proteins were merged to a single gene identifier. The next step to reduce the abundance was connecting all gene identifiers with exactly the same allocated peptides. These gene identifiers were summed up and given the name of the gene identifier with the most allocated peptides or the shortest amino acid sequence by identity. In the end gene identifiers with an overlap of allocated peptides were also combined to one gene identifier. The name of the gene identifier was chosen on the basis of the number of uniquely allocated peptides or the length of the amino acid sequence. All proteins with only one allocated peptide were handled as not significant and are listed in Tables S5–S7 in Supplementary Material.

#### Prediction of outer/inner envelope membrane proteins

All gene identifiers including splice variants and proteins, which could be identified with the allocated peptides were used to predict the envelope membrane proteins. Two different experimental approaches were applied for *P. sativum*. The first approach for mass spectrometry analyses contained purified OE proteins. The other approach contained purified IE proteins. The peptides detected by MS were blasted against a database of contigs and singlets of *P. sativum*. For classification of the detected contigs and singlets to outer or IE proteins, we first had to find orthologs in *A. thaliana*. The contigs of the *P. sativum* database were blasted against the *A. thaliana* protein database and subsequently the best hit was reblasted against the *P. sativum* contigs database to verify the *A. thaliana* protein. The dedicated *A. thaliana* gene identifiers were used for the prediction of the OE and IE membrane proteins. All gene identifiers with at least four assigned peptides were used for the analysis of the membrane protein prediction.

Also the identified gene identifiers were allocated to the sub-compartments in the chloroplasts. For this the Plant Proteome Database (Sun et al., 2009) was used, which includes the experimentally annotated localizations of the *A. thaliana* gene identifiers. In the end, the amino acid sequences of the identified proteins in the envelope pools were used to predict transmembrane α-helices via TOPCONS single^4^ (Hennerdal and Elofsson, 2011).

#### Database comparison

The proteins of the three different organisms detected in our envelope studies were compared to previous envelope studies including proteomic data for the membrane envelope of plastids by Bräutigam et al. (2008), Bräutigam and Weber (2009), Ferro et al. (2003, 2010), and Froehlich et al. (2003). Also the detected proteins are categorized concerning their occurrence in the different studies and stroma or thylakoid in this study or the study of Ferro et al. (2010).

#### Domain and homolog searches, structural predictions

First, the function and the name of the protein represented by the gene identifiers of Tables 3– 6 were looked up in Aramemnon rel. 7.0^5^ (Schwacke et al., 2003). Afterwards, the predicted transmembrane fold was annotated. If Aramemnon predicts transmembrane β-barrel structures the sequences of the gene identifiers were used to build 3D models of respective amino acid sequence with the help of alignments to known protein structures via the protein fold recognition server Phyre2 (Kelley and Sternberg, 2009). For the gene identifiers of unknown function, the putative domains were searched using the *P*rotein *fam*ilies database (Pfam; Finn et al., 2010) and the Conserved Domain Database (CDD; Mitra et al., 2007).

## Conclusion

The determination of subcellular and suborganellar proteomes or alterations thereof (due to, e.g., environmental changes) by mass spectrometry is still limited in respect to protein abundance and sample purity (Figure 1), but most likely not by bioinformatic methods used for protein assignment (Figure A1 in Appendix). The assignment of peptides depends in general on their length and the false positive rate can be regulated by mapping criteria. Unassigned peptides usually observed in such studies can in parts be explained by the stringency of the mapping criteria, but point also toward natural variances at the protein level.

In the study at hand, we performed proteomic analyses of chloroplast envelope membranes from three different plant species. The necessity to sustain proteomic studies on the analyses of different species was formerly shown by the unexpected high diversity of soluble chloroplast proteomes, when comparing data from *A. thaliana* and *P. sativum* (Bayer et al., 2011). The comparison of envelope fractions from different plant species in our study increased the number of detected proteins but did not result in a large intersection of these envelope proteins (Figure 2; Table 2).

Furthermore, when comparing our findings with previous proteomic envelope approaches, we were able to refine the available proteome data and assign a reliable, comprehensive core proteome. Contrary to expectations, intersection of proteins identified in these studies was rather small (Table 1). Altogether, we identified 191 potential envelope proteins (categories I–III). After detecting putative cross-contaminations of stromal and thylakoid proteins the remaining 136 envelope proteins were clustered according to their predicted/confirmed localization and cellular function (Figure 3). To this end 35 IE, 24 OE, and 19 known non-assignable envelope proteins were identified. Amongst these UBQ1 and SUR2 as well as AKR2B, UBQ11, Oep16-2, and Oep24 were newly assigned to IE and OE, respectively.

Moreover, we identified 21 new potential envelope proteins of category III of unknown function which might give rise to further analyses. Finally, we observed differences concerning the predicted localizations in the independent studies which point toward a possible membrane-association or a possible dual or multi-sublocalization inside the chloroplast or cell.

## Conflict of Interest Statement

The authors declare that the research was conducted in the absence of any commercial or financial relationships that could be construed as a potential conflict of interest.

## Supplementary Material

The Supplementary Material for this article can be found online at http://www.frontiersin.org/Plant_Proteomics/10.3389/fpls.2013.00011/abstract

Supplementary Table S1 **Proteins identified in the *A. thaliana* envelope membrane fraction**. The first column gives the AGI number, the second the number of identified splice variants, the third the AGI of the splice variants, the fourth the AGI code of similar proteins detected, the fifth column the number of peptides assigned to the protein only, the sixth column the number of peptides additionally assigned to other proteins, and the seventh column a short description of the protein. In the second sheet the AGI number and all identified peptides are listed. Every peptide is identified by MS/MS.

Supplementary Table S2 **The proteins identified in the *M. sativa* envelope membrane fraction**. The first column gives the AGI number, the second column the *Medicago* specific ID, the third the number of identified splice variants, the fourth the AGI of the splice variants, the fifth the AGI code of similar proteins detected, the sixth column the number of peptides assigned to the protein only, the seventh column the number of peptides additionally assigned to other proteins, and the eight column a short description of the protein. In the second sheet the *Medicago* ID number and all identified peptides are listed. Every peptide is identified by MS/MS.

Supplementary Table S3 **The proteins identified in the *P. sativum* outer envelope membrane fraction**. The first column gives the AGI number, the second column the *Pisum* specific ID, the third the number of identified splice variants, the fourth the AGI of the splice variants, the fifth the AGI code of similar proteins detected, the sixth column the number of peptides assigned to the protein only, the seventh column the number of peptides additionally assigned to other proteins, and the eight column a short description of the protein. In the second sheet the *Pisum* ID number and all identified peptides are listed. Every peptide is identified by MS/MS.

Supplementary Table S4 **The proteins identified in the *P. sativum* inner envelope membrane fraction**. The first column gives the AGI number, the second column the *Pisum* specific ID, the third the number of identified splice variants, the fourth the AGI of the splice variants, the fifth the AGI code of similar proteins detected, the sixth column the number of peptides assigned to the protein only, the seventh column the number of peptides additionally assigned to other proteins, and the eight column a short description of the protein. In the second sheet the *Pisum* ID number and all identified peptides are listed. Every peptide is identified by MS/MS.

Supplementary Table S5 **Peptides identified by analysis of *A. thaliana* fractions not assigned to a protein**. The peptide, the type of digestion yielding the peptide and the fraction(s) the peptide was identified in is given in sheet one. In sheet two the *Arabidopsis* ID, the peptide, the type of digestion yielding the peptide, the fraction(s) the peptide was identified in, and the short description of the protein is given for all proteins identified by a single peptide only. In sheet three the Arabidopsis IDs, the peptide, the type of digestion yielding the peptide, and the fraction(s) the peptide was identified in is given for all peptides leading to the identification of multiple proteins.

Supplementary Table S6 **Peptides identified by analysis of *P. sativum* fractions not assigned to a protein**. The peptide, the type of digestion yielding the peptide, and the fraction(s) the peptide was identified in is given in sheet one. In sheet two the *Arabidopsis* ID, the *Pisum* ID, the peptide, the type of digestion yielding the peptide, the fraction(s) the peptide was identified in, and the short description of the protein is given for all proteins identified by a single peptide only. In sheet three the *Arabidopsis* IDs, the *Pisum* IDs, the peptide, the type of digestion yielding the peptide, and the fraction(s) the peptide was identified in is given for all peptides leading to the identification of multiple proteins.

Supplementary Table S7 **Peptides identified by analysis of *M. sativa* fractions not assigned to a protein**. The peptide, the type of digestion yielding the peptide, and the fraction(s) the peptide was identified in is given in sheet one. In sheet two the *Arabidopsis* ID, the *Medicago* ID, the peptide, the type of digestion yielding the peptide, the fraction(s) the peptide was identified in, and the short description of the protein is given for all proteins identified by a single peptide only. In sheet three the *Arabidopsis* IDs, the *Medicago* IDs, the peptide, the type of digestion yielding the peptide, and the fraction(s) the peptide was identified in is given for all peptides leading to the identification of multiple proteins.

Supplementary Table S8 **List of all identified proteins**. The *Arabidopsis* IDs of all proteins identified in this study including those with only one peptide matching are listed. The first column gives the ID, the second column the predicted compartment the protein is supposed to be localized in, the column 3 the Arabidopsis fraction, columns 6 and 7 the two Pisum fractions, and column 8 the Medicago fraction; the last column indicates whether the protein is identified in at least one fraction by more than one peptide (norm) or whether identification occurred by one peptide match only (onehit). The fraction the protein was identified in is marked by X.

Supplementary Table S9–S12 **List of all proteins in category I**. The first column is the AGI identifier, the second column the name and aliases of the protein, and the third column the number of studies, where the protein was identified. Category Ia are proteins found in our study and at least one other study and category Ib are proteins identified not in our study but at least two other studies. Category IIa are proteins found in our study and at least two other studies but also in the stromal or thylakoid fraction. Category IIb are proteins found in three other studies and also in the stromal or thylakoid fraction. Category IIIa are proteins only identified in our study and category IIIb are proteins found only in one study excluding our study. Category IVa and IVb contains proteins identified in the stromal or thylakoid fraction and only in our and less than two other studies (IVa) or in less than three other studies (IVb).

Supplementary Table S13 **List of overlapping and not overlapping proteins in the Venn diagram**. The first column gives the AGI identifier, the second column the name and aliases of the protein, the third column the number of studies where the protein was identified, the fourth column the category of the protein, the columns 5–9 show in which envelope fractions and plant species the proteins could be identified. X, identified; –, not identified.

Supplementary Table S14 **List of the ions score cutoff and FDR for nLC-MALDI MS/MS**. The first column gives the used MS method, the second column the organism and fraction, the third column the restriction enzyme, the fourth column the used database for searching, the fifth column the number of repetition, the sixth column the ions score cutoff in −10log(*p*) by *p* = 0.05, and the seventh column the false discovery rate (FDR). The used databases are the UniProtKB, the MT3.0 from IMGAG for *Medicago truncatula*, and the EST-library by Franssen et al. (2012) for *Pisum sativum*.

Supplementary Tables S15–S57 **Raw data measured by nLC-MALDI MS/MS**. Each excel sheet is grouped in two levels. The first level contains information for each identified accession ID. The first column gives the accession (UniProtKB, IMGAG, or EST-library by Franssen et al., 2012), the second column the coverage, the third column the number of peptide spectrum matches (#PSMs), the fourth column the number of peptides, the fifth column the number of amino acids (#AAs), the sixth column the molecular weight (MW in kDa), the seventh column the isoelectric point (pI), the eighth column the score, and the ninth column the description. The second level contains all peptide information for each accession ID. The second column gives the confidence icon (Low; Medium; High), the third column the peptide sequence, the fourth column the protein accessions, the fifth column the number of proteins, the sixth column the number of protein groups, the seventh column the activation type (Collision Induced Dissociation, CID), the eighth column the modifications, the ninth column the ion score, the 10th column the expectation value (exp. value), the 11th column the delta score (Δscore), the 12th column the rank, the 13th column the identity High, the 14th column the homology threshold, the 15th column the charge, the 16th column the mass to charge ratio in daltons (*m*/*z*), the 18th column the delta mass (ΔM, difference between the theoretical mass of the peptide and the experimental mass of the precursor ion), the 19th column the matched ions, and the 20th column the spectrum file.

## Footnotes

^1^ftp://ftpmips.gsf.de/plants/medicago/MT_3_0/, International Medicago Genome Annotation Group

^2^ftp://ftp.arabidopsis.org/home/tair/

^3^ftp://ftpmips.gsf.de/plants/medicago/MT_3_0/

^4^http://single.topcons.net/

^5^http://aramemnon.uni-koeln.de/index.ep
